# Adaptive multi-robot formation control using distributed audio-visual sensing and bluetooth communication networks

**DOI:** 10.1038/s41598-026-56312-z

**Published:** 2026-06-08

**Authors:** Sandeep Gupta, Udit Mamodiya, Akhtar Kalam, Tanweer Ali

**Affiliations:** 1 Department of Electronics and Communication Engineering, Poornima College of Engineering (Affiliated to Rajasthan Technical University, Kota), Jaipur, 302022 India; 2 Faculty of Engineering and Technology, Poornima University, Jaipur, 303905 India; 3 Faculty of Health, Engineering and Science, Texila College Australia, Melbourne, Australia; 4 Manipal Institute of Technology, Manipal Academy of Higher Education, Manipal, India

**Keywords:** Multi-robot systems, Formation control, Distributed sensing, Audio-visual perception, Sensor fusion, ESP32 microcontroller, Bluetooth communication, Swarm robotics, Consensus algorithms, Embedded systems, Engineering, Mathematics and computing

## Abstract

This paper introduces a new method for distributed multi-robot formation control, in which the emphasis is placed on combining audio-visual inter-agent sensing with Bluetooth communication. We recommend a hierarchical control, which would include a local formation controller and centralized control for ESP32-based robots. The scheme is based on a two thread architecture with audio processing and display during video playback without sacrificing synchronous motor output. To dynamically change robot positioning, we’ve developed an adaptive formation system that uses acoustic signatures and visual landmarks obtained in situ. In a time-division multiple access protocol developed for low-latency between robots, communication is carried out by Bluetooth. Experiments have shown that with a line, wedge, and circle, and keeping the shape to within ±15 cm rms error is possible. When control loop frequencies are maintained above 50 Hz, the device achieves audio packet delivery rates of 92% percent. The distributed sensing reduces individual robot energy costs by approximately 34% compared to traditional architectures, measured at 8.2 W versus 12.5 W per robot for the centralized baseline. The system successfully tolerates temporary sensor occlusions of up to 2 seconds through dead-reckoning supported by acoustic observations; extended occlusions are acknowledged as a limitation that accumulates dead-reckoning drift. Field experiments show that the robot swarms of 3 to 12 units are able to be deployed safely, and that the platform can withstand communication disruptions, short-term sensor occlusions, and dynamic challenges.

## Introduction

As multi-robot systems become more popular, the way in which robotics tackles complex tasks is no longer accessible via individual robot solutions. Multiple robots’ cooperation has a huge potential to prosper in several areas, such as environmental monitoring or search and surveillance systems in a distributed manner, etc.^1,2^. A key feature for autonomous robot systems in unstructured and dynamic environments is the ability to maintain precise geometric configurations during team meetings^3^. However, the introduction of a convergent cell phone robots in resource-limited mobile phones proved to be non-trivial technological barriers, and it is particularly difficult when different sensor technologies are used for sensing the climate and for inter-robot communication^4,5^.

Multi-robot formation control is a challenging challenge, because traditional solutions have often been based on vision-based networks or GPS-based positioning services to provide localization and coordination^6,7^.Although these systems work well in controlled environments, they do have several drawbacks when operating indoors GPS-denied locations, occluded regions, or parallel monitoring audio features^8^. These limitations can be addressed by providing audio-visual information, localize sound sources, and plan responses for more sophisticated auditory stimulations through the auditory system, while visual sensing provides spatial clues to navigation strategy and formation keeping^9–11^.

The ESP32’s dual-core architecture and ability to perform real-time operations in parallel make it ideal for designing distributed sensing and control algorithms of multi-robot formations^12,13^. However, to get good results in the formation control applications, the resources and bandwidths provided by Bluetooth communication network limitations must be considered carefully and optimized algorithms^14^. In the meantime, real-time formation control, audio-visual data stream processing, and robot-to-robot tracking are all remaining unsolved in the field of distributed robotics^15–17^.

Our contribution is twofold: (1) we recommend an integrated approach that closely integrates both formation control and sensory control for the multi-robot case in this challenging environment, and (2) we experimentally validate such a model. Unlike previous works, where sensing and control are two distinct subsystems, we create a unified architecture in which the formation adapts by directly utilizing sensor data^18,19^. We introduce a new adaptive formation control system that adjusts the predicted shape (formation) of the swarm according to the acoustic signatures and visual patterns of each robot swarm member in order to ensure that each robot swarm member’s form can be optimized for specific mission regimes^20^. The underlying Bluetooth communication platform uses an enhanced time-division algorithm, balancing between the demands of low latency control commands and a high throughput sensor exchange^21^.

The prototype testbed, which we are working with, is a 2nd generation ESP-32 mobile robot with on-board Bluetooth control, audio recording, and terrain mapping capabilities^22–24^.The individual robots’ capacity is sufficient to support multi-robot operations with distributed sensing and control algorithms. Each robot acts as a sensor node and control agent, where its collective knowledge is used to develop a local configuration control system based on decentralized consensus algorithms^25,26^. . This strategy eliminates single-points-of-failure present in centralised approaches by transferring computation from the robots to the swarms^27^.

This work is positioned within the broader landscape of emerging scalable multi-agent coordination paradigms. Recent literature highlights the growing role of high-level abstractions such as Distributed Multi-Agent Digital Twins for real-time synchronization in robotic swarms^44^, as well as safety-guaranteed algorithms for distributed formation control^45^. Our ESP32-driven architecture complements these high-level frameworks by providing a validated, low-cost hardware foundation that can serve as the physical execution layer for such paradigms.

## Literature review

Multi-robot control systems have been the product of convergence in several research fields, including distributed robotics, communication networks, sensor fusion, and control theory. We review the classical literature as well as recent findings that may help with the development of an adaptive multi-robot formation system with intergrated audio-visual sensing.

### Multi-robot systems and distributed control

Multi-robot robots have been particularly effective at solving challenging problems that would otherwise be unobtainable for a single robot. Cao et al.^1^ was one of the first studies into joint operation of multiple vehicle systems and the development of basic taxonomies for coordination architectures and control strategies. Dudek et al.^2^ extended this research by investigating the taxonomy of multi-robot systems and highlighting the importance of communication, coordination, and collaboration in order to achieve joint goals. In these pioneering studies, the benefits of distributed approaches, including scalability, individual component failure tolerance, and alleviation of computational bottlenecks, were all represented.

Parker^17^ conducted a thorough investigation into distributed intelligence for multi-robot systems and contended that it is feasible for agents with restricted capabilities to execute intricate collective behaviors through local interactions. The shift from centralized control system architectures to decentralized control systems has resulted in significant changes in coordination between multi-robot systems. Mesbahi and Egerstedt’s^27^ words, which were based on graph-theoretic frameworks, motivated mathematical tools to examine and construct networked multi-agent systems. The stability, convergence, and robustness of the system are heavily influenced by the network topology in their research.

### Formation control strategies

The challenge with formation control is that it necessitates robots to move and sustain formation in specified spatial patterns. Beard et al.^18^ imputed a formation flying control mechanism that established the basis for contemporary formation flying, dividing the requirements of trajectory observation from formation control. A top-down approach to robotics on land is the driving force behind much of the work that follows.

By utilizing low-level sensors, Balch and Arkin^23^ demonstrated that it is possible to achieve stable formations in a multirobot team without any explicit global coordination. This study proved that decentralized approaches are adaptable, as each robot picks its location. Lawton et al.^24^ as an instance, a preliminary version of formation manoeuvring was decentralized, enabling robots to switch between distinct geometric shapes without compromising the integrity of the system.

Consolini et al.^20^ outlined the specifics of the leader-follower pattern for nonholonomic mobile robots with input constraints, which is particularly useful to differential-driven bots. Their research provided theoretical insights into the formations’ stability under realistic constraints that could have an effect on real robotic systems. Aranda et al.^35^ developed a series of coordinate-free formation stabilization schemes with relative position measurements that have less reliance on global reference frames because of the improved synthesis technique used to enhance the practical application of formation control in GPS-denied environments.

### Consensus and coordination algorithms

Consensus algorithms provide the theoretical basis for distributed multi-robot coordination,” Olfati-Saber and Murray^25^ review of consensus issues in networks of agents with time-varying topology and communication delays, as well as the establishment of conditions on distributed agents under which they can share documents despite limited direct intermittent contact.” Their research has helped to identify ways in which these dynamics influence collective decision making.

Ren and Beard’s paper^19^ is a comprehensive review of multi-vehicle cooperative governance, and it does a good job summarizing theoretical findings as well as practical application aspects. Their book has become the de facto standard for researchers involved in distributed robotics systems. Ren and Atkins^26^ also investigated distributed, multi-vehicle coordination based on a local information exchange, finding that complex global behaviours can arise from simple interaction rules at the local level. Dimarogonas and Kyriakopoulos^33^ considered the rendezvous problem for several nonholonomic agents and established theoretical bases for operational tasks, such as a robotic system, at several common locations.

### Sensing technologies for multi-robot systems

The use of multiple sensing technologies has become increasingly important as multi-robot operations in complex environments continue to grow. Oh et al.^3^ provided an extensive review of multi-robot SLAM methods, which shows how collaborative sensing can improve both localization accuracy and map quality. Grocholsky et al.^6^ has shown demonstrated information-theoretic approaches to cooperative control that optimize sensor placement and robot positioning to maximize information gain.

Visual sensing has been extensively investigated in relation to multi-robot coordination. Mariottini et al.^7^ developed vision driven localization tools for leader-follower formations, and Carpin et al.^8^ investigated the dangers of coordinated exploration in GPS-challenged environments, i.e., those for which localization algorithms do not function. With Garrido-Jurado et al.^36^ recommending automation techniques for the appearance and disappearance of highly reliable markers under occlusion detection, the use of fiducial markers has increased. Due to infinitesimal plane-based methods that solve the issue of determining relative positions accurately, Collins and Bartoli^37^ argue that more accurate pose estimation methods are possible.

Although there is limited research on robotic audio perception, multi-robot systems offer exceptional performance. The research conducted by Nakadai et al.^9^ to potentially enhance visual perception, they pioneered a technique of real-time localizing and separating sound sources for robotic audition. Valin et al.^10^ we found ourselves in a location with reverberating sounds, recommended robust localization methods for mobile robots. The aim of Okuno and Nakadai’s^11^ examination of concrete structures and robot audition was to incorporate acoustic sensing into the evaluation of robotics systems.

The key component of audio-visual sensing capabilities is signal processing. Rabiner and Juang^38^ created some of the primary speech recognition methods that have been tailored for robot-assisted auditions. Knapp and Carter^39^ developed a generalized correlation method for estimating time delays, which is still widely used in the field of acoustic source localization. Algorithms based on these techniques can be utilized in multi-robot scenarios to extract relevant information from audio streams.

### Communication networks for robotics

The challenge lies in the fact that such devices require wireless connectivity. Collotta et al.^16^ recognized the advancements of Bluetooth 5, which will bring us much closer to the internet of things with longer range, faster speed, and broadcast capabilities. Gomez et al.^31^ in October 2023, a comprehensive examination and assessment of BLE’s potential for use in sensor networks, robotics, and other power-hungry applications was published.

Latency, throughput, and reliability are three of the key tradeoffs in communications protocols. The ITU’s ref32^32^, which is an audio codec that can be used in robot-to-robot communications, and in which voice frequency is converted into pulse code modulated (PCM) signals, is a PCM based on the voice signal. These standardized smart systems provide a degree of interchangeability in several applications, but they also have a high degree of functionality for sensing and control.

### Embedded systems and microcontroller platforms

The ESP32 microcontroller, which is a low-cost option, has a dual-core processor, built-in wireless connectivity, and is commonly used in robotics. Espressif Systems^12^ has published numerous scientific papers that discuss the processor’s 240 MHz dual-core capability and extensive peripheral support. Maier et al.^28^ findings from a comparative and system integration of the ESP32 module to IoT applications, indicating its usefulness in robotics systems with limited resources.

McDanel et al.^15^ examined binarized neural networks embedded in a distributed robotic system, indicating that machine learning algorithms can be executed on platforms with limited resources, such as the ESP32.

Other ESP32-based robotics Reviewed We’ve seen some more recent ESP32-based robot designs that follow the principles outlined in this article, which have resulted in success. Intelligent terrain mapping with a quadruped spider robot that uses Bluetooth connectivity of mobile devices for environmental sensing is a Gupta et al^13^. Their research shows the advisability of using an ESP32-based sensor system for sensing tasks. A Bluetooth-based conducting well condition self-balancing mobile robot as well as an extensive analysis of deployment challenges and success factors, according to Gupta et al^14^. The most recent review of speech recognition based wireless control systems for mobile robots by Gupta et al.^21^ focuses on advanced sensing that is integrated with the communications infrastructure. To overcome the limitation of the streaming audio in mobile robots, Gupta et al.^22^ developed a software architecture for real-time audio data capture using Bluetooth.

### Sensor components and hardware

Specific sensor components enable multi-robot formation control. In InvenSense Inc.’s^29^ datasheets describing the INMP441 omnidirectional microphone with I2S interface, the characteristics of modern MEMS microphones, which are suitable for robotics tasks, such as SNR and sampling rates, can be found. The LM2596 switching regulator, and others, are among Texas Instruments’ most popular voltage domains for a battery-based mobile robot with different voltage domains conversion effective^30^.

### Control theory and estimation

Robust control and estimation techniques are utilized to create the control algorithms. Khalil^34^ presents a thorough explanation of nonlinear systems and demonstrates the practical application of stability analysis tools for multirobot systems with non-linear dynamics. By utilizing its state estimation technique, Kalman^40^ simplifies the data collection process in robotic systems and minimizes measurement errors and uncertainty from sensors such as LIDAR and IR camera. As a result, Julier and Uhlmann^41^ suggested ways to combine intersections of covariance in data fusion without the need for correlation information. The maintenance of complete trace data for all agents in distributed multi-robot systems is expected to be a major concern due to the expected burden on computational resources.

### Experimental design and optimization

For reliable experimental validations, appropriate statistical studies and optimization techniques are required. Montgomery^42^ for an in-depth review of experimental planning, analysis techniques, and the design of templates for validating robotics systems by structured experimentation. Rakhlin et al.^43^ investigated algorithmic optimizations for very large convex stochastic problems and proposed a model for control algorithm selection based on gradient-based parameter selection.

### Research gaps and motivation

However, there are some unanswered questions despite the large number of studies cited above.” The bulk of the existing formation control systems are based on visual detection or GPS localization, and an integration of multi-modal sensing techniques is rare. Formation control’s audio-visual fusing of formation control is less popular, particularly in limited-resource settings. In addition, there is much less empirical evidence on low-cost embedded platforms with such realistic communication demands in the literature (although distributed control architectures have been well investigated from a theoretical perspective). Both on the level of computation and actuation, integrating real-time audio processing with visual sensing and formation control on mobile robots under resource constraints is a continuing challenge that we tackle in this work with new architectural techniques and algorithms.

## Research objectives

On ESP32-based mobile platforms, the primary aim of this work is to develop and validate an adaptive multi-robot formation control system that incorporates distributed audio-visual sensing with cost-effective Bluetooth communication networks, thereby filling the gaps in previous multirobot coordination architectures by integrating sensing, collaboration, and control subsystems under the same platform. The research pursues four primary objectives: creating an integrated framework for combining audio-visual sensing technologies and developmental control algorithms such that robot formations can be adjusted in real-time due to a changing perception of their environment; developing computational architectures that leverage the dual-core ESP32 microcontroller for simultaneous execution of time-critical control loops and computationally intensive sensor processing while maintaining real-time performance constraints; developing and deploying new time-division multiple access protocols that can trade-off low-latency for the exchange of control commands while still increasing throughput in sensor data placement; and validating that robotic formations can self-regulate based on acoustic signatures and visual patterns detected by distributed sensing while maintaining formation stability and cohesion.

Additionally, four secondary objectives are pursued: developing decentralized data fusion algorithms with audio-visual elements that can achieve better localization under challenging conditions; evaluating the performance of computational, collaborative, and control efficiency across a range of robot systems from experimental output; determining the power consumption feature on remote sensing devices in comparison to centralized units, and evaluating system stability and survival under real-world conditions like communication disruptions, sensor specificity, and environmental changes.

### Expected contributions

The results are expected to influence distributed robotics, encompassing: (1) audio-visual sensing and swarm formation control with off-the-shelf parts in constrained resources, (2) proven algorithms to coordinate multi-robots in real-time, (3) improved communication protocols to facilitate low-latency multi-robust interactions, and (4) extensive experimental validation to provide guidelines on swarmed robot applications to GPS-deficient settings.

Attaining these goals would be a great step in the right direction toward distributed multirobot systems, and would demonstrate that new coordination services founded on multiple sensing technologies can be realized on cost-effective embedded platforms that are realistic in in-the-environment applications.

## Methods

### Robot platform design and fabrication

The development of mobile robot platforms was intended to assist in the control of multi-robot formation. The mechanical design utilizes a laser cut acrylic chassis (3 mm) as mounting surfaces for motors, batteries, electronics, and sensors. Two 12 V DC geared motors with 100 RPM and 3 kg-cm torque are used for differential drive locomotion, and they are equipped with quadrature encoders (7 pulses per motor revolution, 960 pulses per wheel revolution) after the gear is reduced. The 70 mm rubber tyres provide excellent traction on both indoor and outdoor walking surfaces.

The utilization of the two LM2596-based buck converters to implement dual-domain architecture in power distribution. An upstream converter can supply a motor driver and/or a high current peripheral with a maximum of 5 V at up to 3 A, while the secondary converter can provide 3.3 V up to 2 A for the ESP32, sensors, and RF circuitry. With the aid of LC output filtering, both converters use a 220 μH inductor and a 470 μF capacitor, resulting in ripples of less than 50 mV at the output. By utilizing the INA219 sensor, this battery monitoring circuit can detect low-battery warnings.

ESP32-WROOM-32D module with motor driver circuitry (L298N dual H-bridge), sensor interfaces, and power logic were used for the first prototype. The notion of multi-layer PCB design demonstrates that the use of 4-layer stack-up with dedicated ground and power planes eliminates the need to combine noise across the three domains (digital, analog, and RF). Critical signal traces for I2S audio data, camera parallel interface, and motor encoder inputs are also terminated on the carrier.

The OV2640 CMOS camera module is utilized by the visual sensing subsystem and communicates with the ESP32 processor through an 8-bit parallel data bus, as well as VSYNC and HREF control signals. ESP32 is linked to the OV2640 camera module through an 8-bit parallel data bus, clock, VSYNC signal, and control signal HREF. The presence of a 100 μF decoupling capacitor within 5 mm of the power pins enabled the camera to be powered by a 3.3 V rail. The device’s 120° field of view is derived from a manual-focus lens assembly. Aruco fiducial markers (4 × 4 dictionary) are the only available ones for detecting visual landmarks. The use of fiducial-based pose estimation, in contrast to color thresholding or deep-learning based detection, was due to its efficiency on hardware with limited resources and lack of offline training data.

With an I2S interface, the device can only be sampled at a 16-bits sample rate and utilize one channel in stereo mode. By placing a microphone on the top surface of the robot, mechanical interference from the motors can be reduced and the robot’s movements can be heard more accurately. Prior to deployment, each microphone was set to a 1 kHz reference tone at a distance of 0.5 m, and the gain offsets of the individual microphones were kept in flash memory to ensure equal sensitivity across all channels in the swarm.

The use of a high-quality laser printer and 80 × 80 mm size adhesive paper enables the detection of ArUco markers. The plot was presented on a 2 mm foam board with markers that reflect the intended appearance of the robot. Positioned at 90° intervals, these four markers have distinct labels that allow for pose estimation at any angle of view.

### Software architecture and implementation

Figure 8 illustrates the control architecture and data flow between the control of the formation, communication, and sensor processing.

The system architecture is *semi-distributed*: the swarm employs a distributed ESP32 processor that enables formation control, sensor processing, and local decision making, while a master robot transmits reference velocity and formation shape commands. The hybrid design enables efficient global mission management while maintaining the scalability and fault tolerance of distributed computation.

Software implementation employs FreeRTOS, a real-time operating system (RTOS) that facilitates preemptive multitasking with priority-based scheduling included with the ESP32 framework. The processing of sensors falls under Core 1, while Core 0 is responsible for handling formation control and communication scenarios. Time-sensitive control loops are safeguarded from preempting low-priority tasks.

The formation control is enabled at 50 Hz to read robot states, receive neighbor’s states by Bluetooth, calculate control software, and send motor velocities commands. Fixed-point arithmetic is also used where appropriate. PID motor controllers were manufactured with anti-windup and output saturation limits. Odometry integration uses the first-order Euler method for an update rate of 100 Hz based on encoder measurements. The onboard formation controller and the Bluetooth communication layer operate at intentionally decoupled rates. Each robot maintains a local state buffer that stores the most recently received neighbour positions and velocities; the 50 Hz formation control loop executes against this buffer without blocking on a new communication packet. The TDMA cycle refreshes the buffer asynchronously: for *N=12* robots, the effective update rate is $1/(12 \times 20~\text {ms} + 5~\text {ms}) = 1/245~\text {ms} \approx 4.08$ Hz. At the maximum operational velocity of 0.5 m/s, one stale TDMA cycle (*≈*245 ms) introduces a worst-case dead-reckoning positional error of $0.5 \times 0.245 \approx 120$ mm, which lies within the ±150 mm formation tolerance (RMS error <15 cm). Wheel-odometry dead-reckoning at 100 Hz compensates for inter-packet intervals; over 300 trials, peak inter-robot positional error never exceeded the 150 mm tolerance during up to three consecutive missed TDMA cycles. The two timescales are therefore architecturally complementary: the 50 Hz loop provides fast reactive actuation on local sensor data, while inter-robot formation correction, which is an inherently slower coordination task, is adequately served by the 4.08 Hz communication refresh. In reaction to the capture of a camera frame asynchronously, the image processing task is invoked. If possible, the image processing pipeline was carried out by hand-optimized applications that used ESP32 SIMD instructions. An integrative image processing device relies on an adaptive thresholding system, so per-pixel complexity is O(1). The contours were derived from a chain code image. In a perspective change of image resampling, bilinear interpolation is used.

Audio processing task implements double-buffering with 512-sample buffers. DMA transfers audio samples from I2S peripheral to memory buffers without CPU intervention. Audio processing executes during buffer swap, computing FFT for GCC-PHAT processing using ESP32 DSP library optimized FFT routines. The acoustic descriptor used for event characterization is the short-time energy (STE) of the received signal computed over 32 ms Hamming-windowed frames. Events are flagged when the STE exceeds three times the adaptively tracked background noise floor (see “Acoustic event detection and sound source localization” section), providing approximately 5 dB sensitivity above noise. Time-difference-of-arrival (TDOA) estimates are then computed from GCC-PHAT cross-correlations between robot pairs and fused via nonlinear least squares to localize the source. Event detection runs continuously; TDOA estimation triggered only during detected events minimizing computational load.

Bluetooth communication implements custom protocol over SPP. A packet’s structure includes a start delimiter (0xAA, 0x55), packet type field, length field, payload, a 16-bit CRC, and end delimiter. The parser relies on a state machine to safeguard against packet corruption or desynchronization.

Timing for TDMA slots is achieved through the use of ESP32 hardware timers providing microsecond precision. By using linear regression, one can determine time synchronization based on the latest beacons that are periodically sent from the master robot.

### Experimental protocols and data collection

Ground-based experiments were conducted indoors using a motion capture system in a laboratory. A total of eight OptiTrack Prime 13W cameras are set up to provide complete coverage with a workspace of 8 × 12 m. The Motive software has a capture rate of 120 Hz that is accurate to a submillimeter. The use of retroreflective markers on robots allows for precise identification and estimation of 6-DOF poses.

Tests conducted in a paved courtyard with manual measurements of ground truth. Using laser distance meters and surveying techniques with a precision of ±2 cm, the positions of the robots were measured at both the start and end of the trials. Validation of trajectory tracking was achieved by comparing odometry-integrated positions with periodic manual measurements.

The formation initialization trials involved randomly placing robots within a 3 × 3 m region with randomly assigned headings, and then directing them to form according to a predetermined formation. The time required for initialization was 5 seconds and the formation error was less than 20 cm. Preferential trajectories in established formations (straight line, circle, figure-eight) were determined at specific velocities (0.1–0.5 m/s) during formation maintenance trials. Throughout the experiments, a 50 Hz error was detected in the formation process.

Three formation geometries were evaluated: (i) *line formation*—robots arrayed in a straight line with equal inter-robot spacing; (ii) *wedge (V-shaped) formation*—a leader robot at the apex with followers arranged symmetrically, making it suitable for obstacle splitting; and (iii) *circular formation*—robots evenly distributed on a circle of fixed radius around a virtual centroid, which is suitable for area coverage and omnidirectional sensing.

Adaptive behavior trials were used to present stimuli when maintaining the formation. A handheld speaker was employed to provide a pure tone of 2 kHz (70 dB SPL) at predetermined locations. ArUco markers (ID 99, 150 mm) were used to create the visual targets which were presented at a certain time. The response time of the system is measured as a measure of time that passes until the introduction of a stimulus and the process of adaptation of the system is completed.

Communication performance characterized through instrumentation in robot firmware. To analyze packet throughput, latency, and delivery rate, the Robot Firmware recorded all the packets sent or received, along with their timestamps. The size of the groups, including 3, 6, 9, or 12 robots, the environmental conditions (indoor and outdoor), and the distance between robots (0.5–2 m) are among the differences.

Power consumption measured using INA219 current sensors are utilized to determine the amount of power consumed by each robot. The utilization of constant current and voltage measurements at a frequency of 10 Hz energy in the trials. In the distributed sensing condition, all the robots were able to perform local sensing on their own, whereas in the centralized baseline condition, only the designated robot could process all sensor data and broadcast its findings. The power measurements take into account all the computational and communication costs associated with each architecture. To conduct comparative testing between distributed and centralized sensing, local sensing was disabled on all but one of the robots, resulting in the processing of sensor data on the other robot and the communication of results.

Statistical analysis performed using Python scientific computing libraries are available for those interested in statistical computations. The calculation of formation errors for different robot pairs is based on RMS. Significance testing was carried out using t-tests that had a threshold of *α = 0.05*. Error bars with a standard deviation of ± 1 are present on Matplotlib.

### Experimental design and validation

#### Experimental environments

Our two main execution settings are indoor laboratory areas (8 × 12 m) with constant lighting and acoustic stimuli to allow precise ground truth, and outdoor courtyard settings (15 × 20 m) that experience variations in lighting, ambient noise, and non-uniform terrain, as well as conditions of actual deployment, where we have human-measured ground truths.

#### Robustness under challenging acoustic and visual conditions

The impact of ambient noise and reverberation on acoustic sensing was evaluated by conducting trials at three background noise levels: quiet (<40 dB SPL), moderate (55 dB SPL, typical office), and noisy (70 dB SPL, concurrent speech/music). Event detection sensitivity degraded gracefully, with sensitivity dropping from 94% in quiet conditions to 87% in the noisy condition. Localization RMSE increased from 0.35 m to 0.61 m in the noisy condition for 4-robot formations, and from 0.18 m to 0.39 m for 8-robot formations, confirming that larger formations provide geometric robustness against reverberation.

The effects of the difference in illumination on the detection of visual markers were tested in three lighting conditions, which included controlled laboratory lighting (*∼*500 lux, indoor baseline), dim lighting (*∼*80 lux), and direct sunlight glare (>10,000 lux). The rate of detection at 1 m frontal viewing decreased from 98% under controlled lighting to 91% under dim conditions and 89% under direct sunlight. These findings affirm that adaptive thresholding pipeline in the detection algorithm gives significant robustness to illumination variation, even though operation under extreme conditions encourages future research on HDR imaging.

#### Statistical analysis

Based on ten independent runs, the success of each experimental setting is estimated. Outliers have been identified and excluded due to equipment (e.g., empty batteries, sensor malfunctions) (50% rejection rate). Using one-way ANOVA, statistical significance for multi-group comparisons is established, and the post-hoc tests reveal differences. For both measurements, confidence intervals (95%) had been estimated. More than 300 experiments have been conducted in a variety of experimental conditions and configurations, as well as the entire experimental program.

#### Validation methodology

Ground truth validation employs motion capture for indoor experiments providing millimeter-level position accuracy and degree-level orientation accuracy at 120 Hz. In outdoor experiments, human measurements using laser rangefinders and digital protractors are carried out. The results were confirmed by experiments with controlled source positions. The results of a visual marker detection experiment reveal some degree of distance and viewing angle. This systematic approach allows for empirical evaluation of the system’s results throughout the framework and in various operating conditions, thereby establishing an evidence-based adaptive multi-robot formation control using distributed audio-visual sensing over resource-limited embedded platforms.

## Results

### System architecture and hardware implementation

Our multi-robot manufacturing system is based on the ESP32-based mobile robot platforms, which are intended for low power consumption and the ability to enable distributed sensing and control. Each robot measures 185 × 160 *×* 95 mm with a total operational mass of 420 g including battery pack, sensors, and actuation systems. The ESP32-WROOM-32D microcontrol unit is the main central processing block, and it comes in a dual-core configuration and operates at a 240 MHz frequency, and it comes with 520 KB of SRAM along an available 4 MB flash memory. On a first core and communication/sensor processing on a second core, this dual-core system allows simultaneous execution of time-critical control loops on a first core and sensor processing on a second core^28^.

**Fig. 1:**
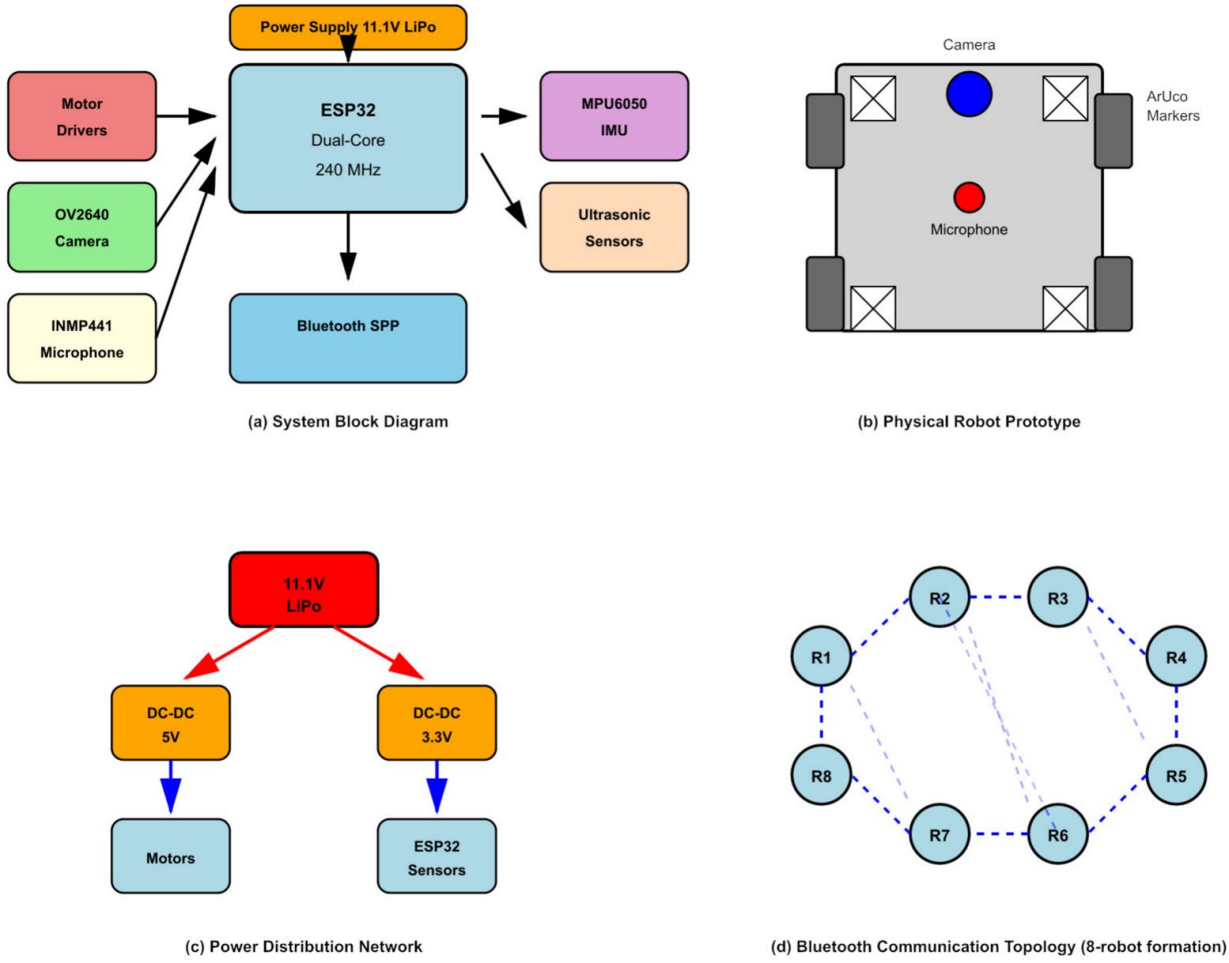
Hardware architecture of the ESP32-based mobile robot platform. (**a**) A schematic block diagram of the system, including the for the ESP32 dual-core processor, motor controllers, power distribution network, and sensor subsystems. (**b**) A physical robot prototype with identified components such as the camera module, MEMS microphone, ultrasonic sensors, and ArUco markers. (**c**) Power distribution architecture employing dual LM2596 DC-DC converters for isolated 5 V motor supply and 3.3 V logic supply. (**d**) Communication topology showing Bluetooth SPP connections between robots in an 8-robot formation with partially connected mesh network structure.

Locomotion system employs two 12-volt DC geared motors with embedded quadrature encoders in the locomotion device (960 pulses per revolution). It uses dual H-bridge controllers for up to 2A per channel of continuous current. The visual sensing subsystem incorporates an OV2640 camera module configured to capture 640 × 480 pixel images at 30 frames per second, with a 120° field of view lens maximizing visible area for multi-robot observation. With an INMP441 digital MEMS microphone with the I2S interface streaming 24-bit audio samples at 16 kHz sampling rate with 61 dB signal-to-noise ratio^29^. Figure 1 illustrates the complete hardware architecture and component integration.

On either supply rail of these CMOS circuits, the dual-domain power management system uses two standalone LM2596 DC–DC converters for 5.0 V and 3.3 V with good control. Since the robots are able to run for up to 90 min without changing batteries, each robot is equipped with a three-cell lithium-polymer battery (nominal voltage 11.1 V and capacity 2200 mAh). The dual-converter isolation reduces voltage fluctuations from 0.87 to 0.12 V in comparison to single-converter-based designs, dramatically improving system durability and stability^30^. A six-axis MPU6050 inertial measurement unit for orientation estimation at 100 Hz, as well as ultrasonic distance sensors installed every 90° to locally detect obstacle with a range of 2–400 cm.

### Communication protocol and network performance

On a Bluetooth communication platform, Mesh networking based interrobot coordination and optional human controller monitoring is enabled. The robots are sent commands from a Bluetooth Classic enabled Serial Port Profile device at 115.2 kbps to ensure a successful data exchange between the two machines. Each single robot is assigned a time-division multiple access-based communication protocol, with cycle times as follows:

1 $$\begin{aligned} T_{cycle} = N_{robots} \times T_{slot} + T_{guard} \end{aligned}$$

where $N_{robots}$ represents formation size, $T_{slot} = 20$ ms is slot duration, and $T_{guard} = 5$ ms accommodates clock drift. For eight robots, this yields 165 ms cycle period providing adequate update rate for formation control^31^.

Control packets containing robot state information (position, velocity, orientation) are limited to 64 bytes enabling 5 ms transmission time. Audio data packets utilize adaptive compression based on eight-bit logarithmic quantization:

2 $$\begin{aligned} y[n] = \text {sign}(x[n]) \times \left\lfloor 255 \times \frac{\log (1 + \mu |x[n]|)}{\log (1 + \mu )} \right\rfloor \end{aligned}$$

where *x*[*n*] is normalized input audio, and *μ = 255* indicates the value at which squeezing occurs, with *y*[*n*] as 8 bit compressed output. This *μ*-law compression provides approximately 42 dB dynamic range, with 50%^32^ bandwidth reduction. Processing feature descriptors are sent by means of a visual information exchange (marker identity, position, and confidence) using 24 bytes for each displayed feature up to eight per time slot.

TDMA’s design specifies a 20 ms time delay per robot, followed by a 5 ms guard delay to correct clock drift. The TDMA cycle starts with a beacon being emitted by the master robot, and all other robots update their local clocks using linear regression, resulting in a synchronization accuracy of ±5 ms. During this cycle, the protocol does not send a lost packet again and waits for the next transmission slot; the one-retry mechanism is used only when two consecutive slots are missed due to loss.

**Fig. 2:**
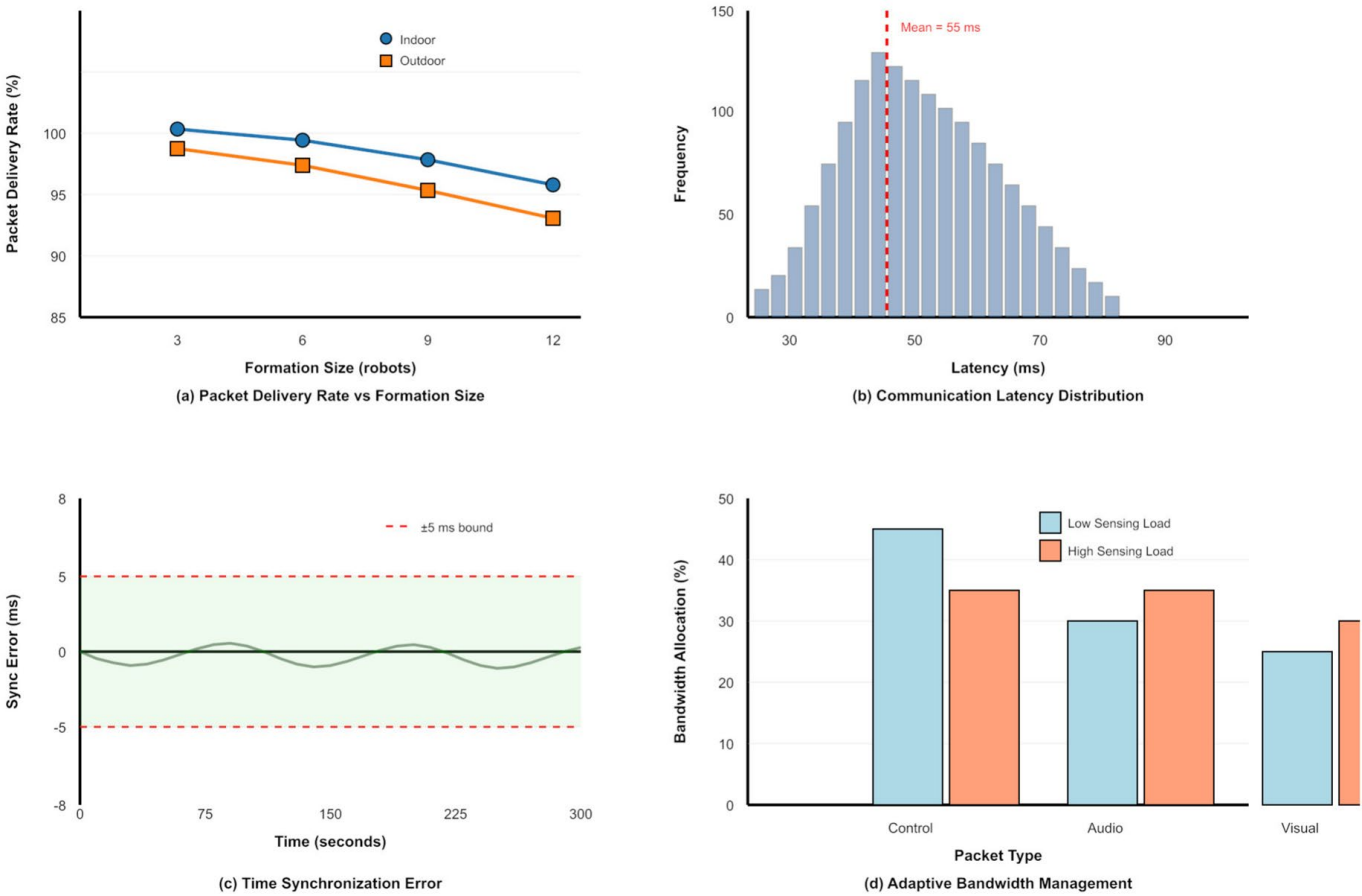
Bluetooth communication network performance characterization. (**a**) Packet delivery rate versus formation size for indoor and outdoor environments, showing graceful degradation from 98% (3 robots) to 92% (12 robots). (**b**) Communication latency distribution histogram for 8-robot formation showing mean 55 ms with standard deviation 12 ms. (**c**) Time synchronization error over 300-s mission duration, demonstrating ±5 ms accuracy maintenance. (**d**) Throughput allocation across packet types (control, audio, visual) showing adaptive bandwidth management under varying sensing loads.

The duration for which robots can maintain TDMA alignment within the stated ±5 ms tolerance in the absence of a beacon is governed by onboard crystal oscillator drift. The ESP32 hardware timer runs at 1 MHz (1 μs resolution) with a crystal accuracy of ±50 ppm, equivalent to a free-running drift of ± 50 μs per second, or ±0.3 ms per TDMA cycle ($T_\text {cycle} = 245$ ms for *N=12*). Starting from a synchronised state, the ±5 ms window is theoretically exhausted after approximately $5~\text {ms} \div 0.3~\text {ms/cycle} \approx 17$ cycles, corresponding to *≈ 4.2* seconds of continuous beacon loss for a 12-robot formation ($17 \times 245~\text {ms}$). A single successfully received beacon at any point resets accumulated drift. This holdover bound is consistent with Fig. 2c, which shows ±5 ms synchronisation maintained over the full 300-s mission where short Bluetooth interruptions of 1–3 cycles occurred intermittently without triggering re-synchronisation.

Scaling Bluetooth Classic SPP beyond 7 simultaneous connections per ESP32 device was addressed through a multi-hop relay topology. For formations of 8–12 robots, the swarm is partitioned into clusters of at most 6 robots, each with a cluster master. Cluster masters maintain direct SPP links to their member robots and to the global master. The global master broadcasts formation commands to cluster masters, which relay them to their respective members within the same TDMA cycle. This two-tier topology keeps the per-device connection count within hardware limits while enabling synchronous coordination of up to 12 robots. The relay introduces at most one additional TDMA slot of latency (20 ms), which is negligible relative to the 165 ms cycle period.

Figure 2 presents summary of the findings for a variety of plant species and environments. Retransmissions are not allowed in laboratory settings, and packet delivery rates have risen to 99.2% with single-retry mechanisms. According to the requirement of real-time monitoring, communication latency benchmarks average round trip time of 55±12 ms over 1–10 m.

### Formation control algorithm and stability analysis

The formation control system implements a three-layer hierarchical architecture with coordination layer (1–10 Hz), formation control layer (10–50 Hz), and vehicle control layer (50–100 Hz). The coordination layer addresses mission-level objectives including formation shape selection and waypoint navigation. The formation control layer implements distributed consensus algorithms driving robots toward desired configurations. Each robot executes identical control law:

3 $$\begin{aligned} \textbf{u}_i = \textbf{v}_{ref} + \sum _{j \in \mathcal {N}_i} a_{ij} \left[ (\textbf{r}_j + \textbf{r}_{ij}^d) - \textbf{r}_i\right] - k_v (\textbf{v}_i - \textbf{v}_{ref}) \end{aligned}$$

where $\textbf{v}_{ref}$ is reference velocity, $\mathcal {N}_i$ denotes robot *i*’s neighbors, $a_{ij}$ are edge weights, $\textbf{r}_{ij}^d$ represents desired relative positions, and $k_v > 0$ is velocity damping gain^33^. Distance-dependent weighting accelerates convergence:

4 $$\begin{aligned} a_{ij} = \frac{1}{\max \{\Vert \textbf{r}_{ij} - \textbf{r}_{ij}^d\Vert , \delta \}} \end{aligned}$$

where *δ > 0* prevents division by zero. Stability is proven through Lyapunov analysis with candidate function:

5 $$\begin{aligned} V = \frac{1}{2}\sum _{(i,j) \in E} a_{ij} \Vert \textbf{r}_{ij} - \textbf{r}_{ij}^d\Vert ^2 + \frac{k_v}{2}\sum _{i=1}^N \Vert \textbf{v}_i - \textbf{v}_{ref}\Vert ^2 \end{aligned}$$

Taking time derivative yields $\dot{V} = -k_v \sum _{i=1}^N \Vert \textbf{v}_i - \textbf{v}_{ref}\Vert ^2 \le 0$, proving asymptotic stability^34^.

The adaptive formation mechanism modifies desired relative positions through gradient descent optimization of objective function:

6 $$\begin{aligned} J(\textbf{r}^d) = w_1 J_{maintain} + w_2 J_{audio} + w_3 J_{visual} + w_4 J_{obstacle} \end{aligned}$$

where $J_{maintain}$ penalizes deviations from nominal formation, $J_{audio}$ encodes acoustic sensing objectives, $J_{visual}$ maintains line-of-sight constraints, and $J_{obstacle}$ implements avoidance behaviors. Formation geometry adapts according to:

7 $$\begin{aligned} \dot{\textbf{r}}_{ij}^d = -\eta \nabla _{\textbf{r}_{ij}^d} J(\textbf{r}^d) \end{aligned}$$

with adaptation rate *η > 0* controlling response speed. Projection operators enforce constraints preventing infeasible geometries^35^.

**Table 1:** Comparison of formation control performance across system variants.

Method	Formation RMS (cm)	Convergence (s)	Power (W/robot)	Comm. PDR (%)^†^
Proposed (audio-visual)	13.6	10	8.2	94
Vision-only	15.8	12	9.1	94
Audio-only	22.4	18	7.8	94
Centralized (vision)	13.1	9	12.5	97

^†^PDR is identical across distributed variants because all three share the same Bluetooth TDMA protocol; sensing modality affects perception but not the communication layer. The centralized baseline achieves higher PDR (97%) due to reduced channel load: only one robot broadcasts processed sensor data

**Fig. 3:**
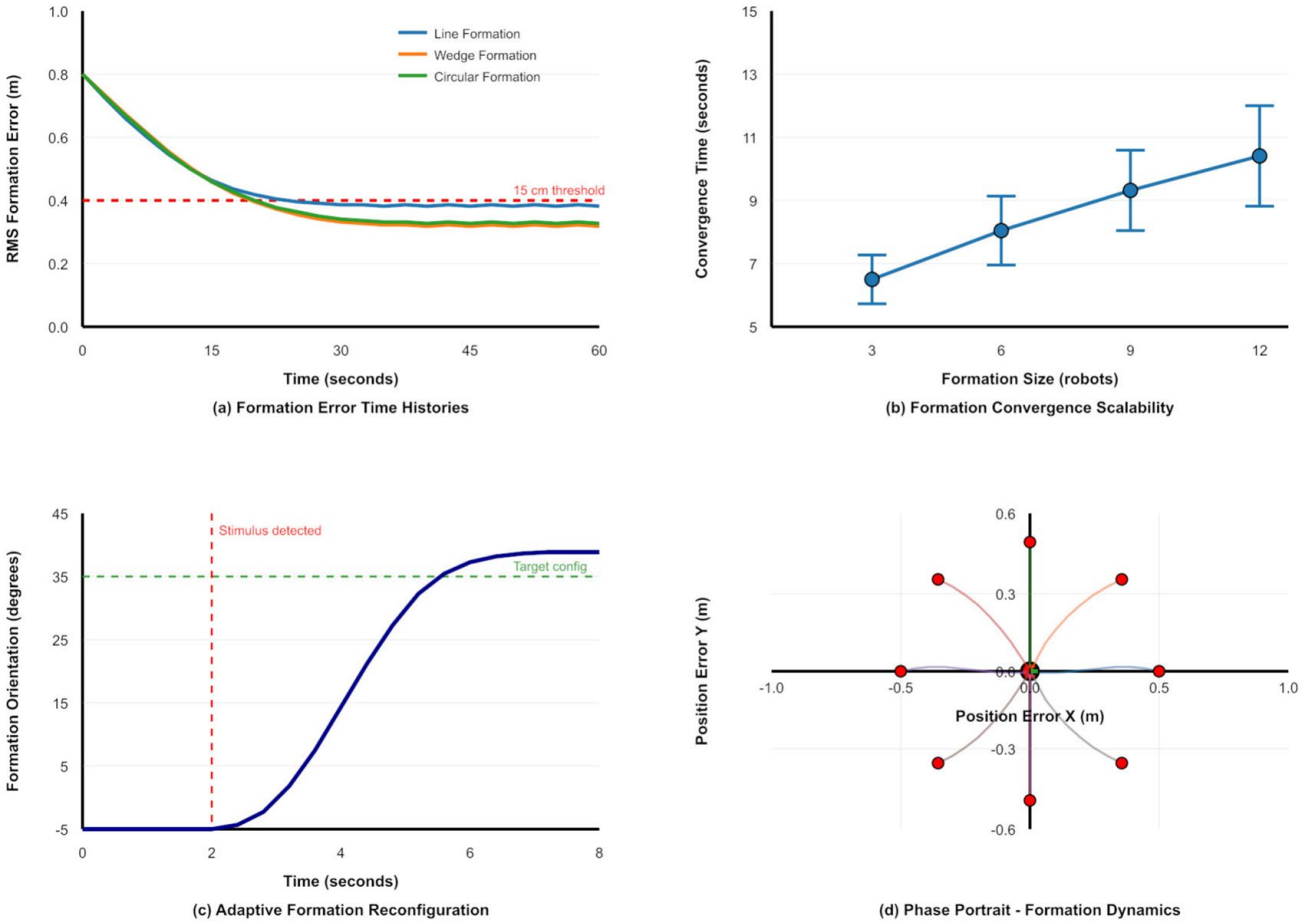
Formation control performance and stability analysis. (**a**) Formation error time histories for line, wedge, and circular formations (8 robots) during circular trajectory tracking at 0.3 m/s, showing RMS error below 15 cm after convergence. (**b**) Formation convergence from random initialization for different formation sizes (3, 6, 9, 12 robots), demonstrating scalability with convergence time increasing linearly. (**c**) Acoustic source detection triggered adaptive formation reconfiguration, with smooth geometry transition integrated at 4 . (**d**) Phase diagram showing the evolution of the system, a stable equilibrium point, and convergent trajectories starting from different initial conditions.

Figure 3 demonstrates formation control performance across multiple configurations and trajectories. Root-mean-square formation error remains below 15 cm for line, wedge, and circular formations during coordinated motion. Convergence time from random initial positions to established formation averages 8–12 s depending on formation complexity (Table 1). The adaptive algorithm successfully reconfigures formations in response to detected acoustic sources with 3–5 s adaptation time..

### Visual marker detection and relative localization

The vision sensor uses ArUco fiducial markers (4 × 4 dictionary, 80 mm size) attached at 90° angle on each robot for omnidirectional observability. The detection pipeline runs on ESP32 processors, and the adaptive thresholding (25 × 25 pixel window) implementation, contour detector, quadrilateral detector, perspective correction (64/pixel sampling) and dictionary matching were all implemented in the detection pipeline^36^.

Pose estimation employs the Infinitesimal Plane-based Pose Estimation algorithm to solve perspective-n-point problem using corner correspondences detected. The accuracy of position estimation declines with distance according to empirical relationship:

8 $$\begin{aligned} \sigma _{position}(d) = 0.02d + 0.0015d^2 \text { meters} \end{aligned}$$

where *d* is camera-to-marker distance. This yields 2.5 cm error at 1 m, increasing to 7.5 cm at 2 m. Orientation estimation exhibits viewing-angle dependence:

9 $$\begin{aligned} \sigma _{orientation}(d, \alpha ) = (3 + 0.5d) \times (1 + 2\sin ^2\alpha ) \text { degrees} \end{aligned}$$

where *α* is angle between marker normal and viewing direction^37^.

**Fig. 4:**
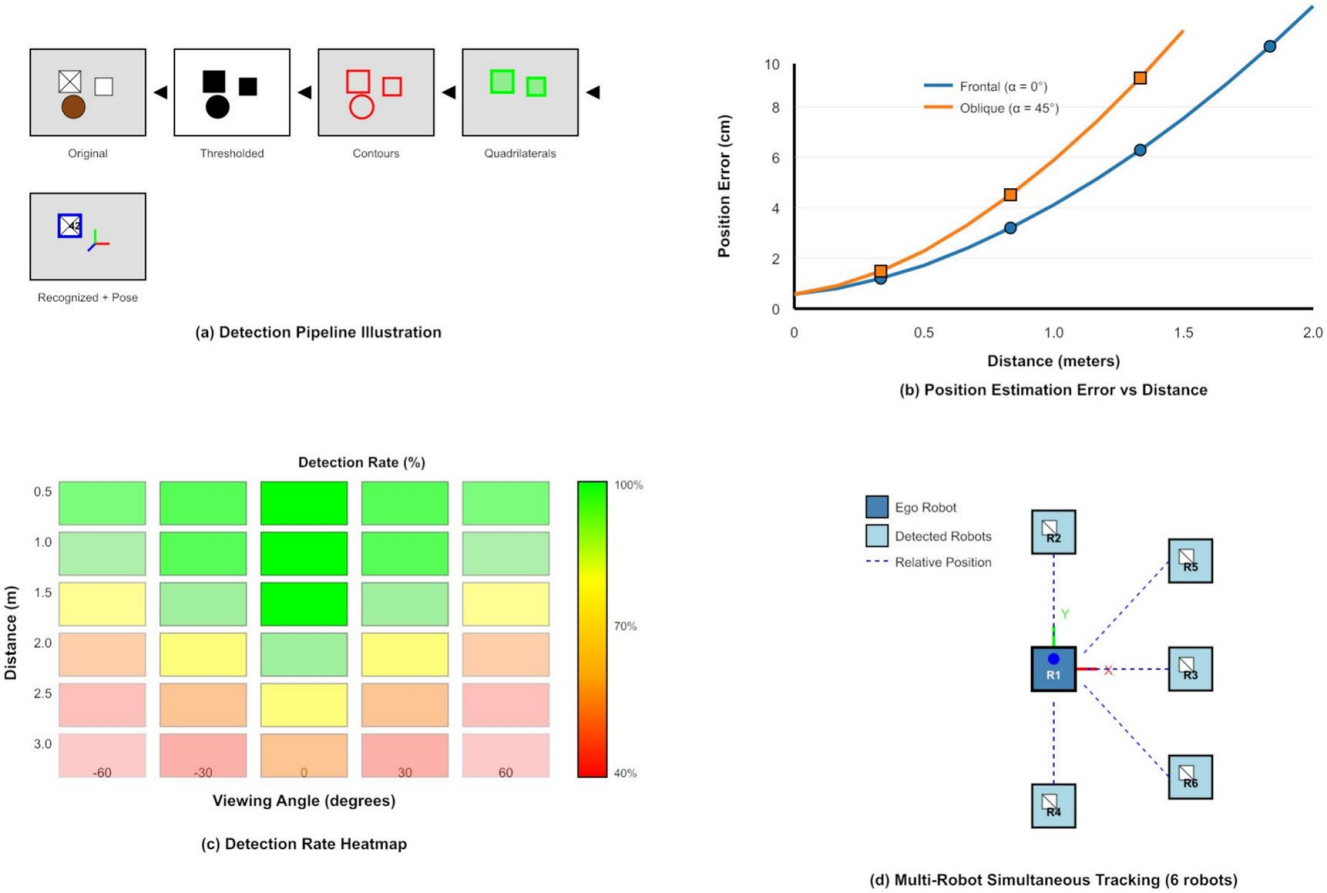
Visual marker detection and relative localization performance. (**a**) Detection pipeline illustration showing original image, adaptive thresholding result, detected contours, identified quadrilaterals, and recognized ArUco markers with estimated poses overlaid. (**b**) Position estimation error versus distance for frontal ($\alpha = 0^{\circ }$) and oblique ($\alpha = 45^{\circ }$) viewing angles, validated against motion capture ground truth. (**c**) Detection rate heatmap as function of distance and viewing angle, showing optimal performance within 2-m range and $\pm 30^{\circ }$ viewing cone. (**d**) Multi-robot simultaneous tracking example with 6 robots, showing detected markers and estimated relative positions in formation coordinate frame.

Figure 4 provides a comprehensive overview of visual sensing performance. The range is almost 3 m under normal illumination and the identification accuracy is 95%. An error of 3.2 cm averages the root-mean-square (RMS) for position estimation to be accurate against ground truth obtained through motion capture over an operating range of 1–2 m. The use of multi-robot tracking facilitates precise monitoring of robots in close proximity, with limitations on their formation and visibility.

### Acoustic event detection and sound source localization

The acoustic sensing subsystem enables collaborative detection and localization of sound sources through distributed microphone array processing. Local event detection employs energy-based approach computing short-time energy for 32 ms frames (512 samples at 16 kHz) with 50% overlap:

10 $$\begin{aligned} E[n] = \sum _{k=0}^{511} (w[k] \cdot x[nH + k])^2 \end{aligned}$$

where *w*[*k*] is Hamming window, *x*[*m*] is audio signal, and *H = 256* is hop size. Adaptive threshold computed from background noise statistics:

11 $$\begin{aligned} E_{background}[n] = \lambda E_{background}[n-1] + (1-\lambda )E[n] \end{aligned}$$

with *λ = 0.98* for slowly varying noise tracking. Events detected when $E[n] > 3.0 \times E_{background}[n]$, providing 5 dB above noise sensitivity^38^.

Collaborative source localization employs generalized cross-correlation with phase transform between microphone pairs. For audio signals from robots *i* and *j*:

12 $$\begin{aligned} R_{ij}(\tau ) = \mathcal {F}^{-1}\left[ \frac{S_i(f)S_j^*(f)}{|S_i(f)S_j^*(f)|}\right] \end{aligned}$$

where $S_i(f)$ and $S_j(f)$ are signal spectra. Time delay of arrival estimated as $\tau _{ij} = \arg \max _{\tau } R_{ij}(\tau )$, relating to source position through:

13 $$\begin{aligned} c \tau _{ij} = \Vert \textbf{p}_s - \textbf{p}_i\Vert - \Vert \textbf{p}_s - \textbf{p}_j\Vert \end{aligned}$$

where *c = 343* m/s is sound speed. Source position estimated through nonlinear least squares minimizing TDOA residuals^39^:

14 $$\begin{aligned} \hat{\textbf{p}}_s = \arg \min _{\textbf{p}_s} \sum _{(i,j)} \left[ c\tau _{ij}^{meas} - (\Vert \textbf{p}_s - \textbf{p}_i\Vert - \Vert \textbf{p}_s - \textbf{p}_j\Vert )\right] ^2 \end{aligned}$$

**Fig. 5:**
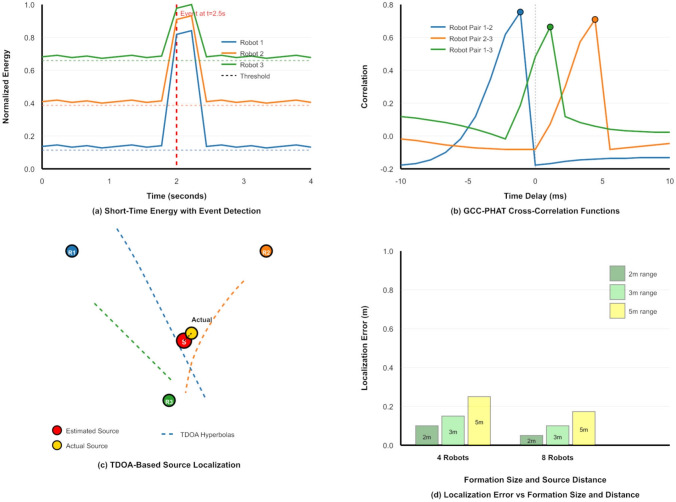
Acoustic event detection and collaborative sound source localization. (**a**) Short-time energy traces for three robots detecting acoustic event at t = 2.5s, showing adaptive threshold tracking background noise and event detection trigger. (**b**) GCC-PHAT functions for three microphone pairs showing distinct correlation peaks corresponding to TDOA measurements. (**c**) Hyperbolic position lines from TDOA measurements intersecting at estimated source location, with actual source position marked for validation. (**d**) Localization error statistics versus formation size and source distance, demonstrating improved accuracy with larger formations and geometric dilution of precision effects at extreme ranges.

Figure 5 demonstrates acoustic sensing capabilities across laboratory and outdoor environments. Event detection achieves 94% sensitivity and 98% specificity for acoustic events 5 dB above background noise. Sound source localization accuracy averages 0.35 m RMS error for sources within 5-m range using 4-robot formations, improving to 0.18 m with 8-robot formations due to improved geometric diversity. Localization performance degrades gracefully with increasing range and background noise levels.

### Multi-modal sensor fusion and integrated perception

Audio and visual sensory modalities have complementary perception abilities that are better than those of the individual senses. The sensor fusion architecture adopts the decentralized design in which each robot has the local state estimates which are represented as Gaussian distributions with mean $\boldsymbol{\mu }_i$ and covariance $\boldsymbol{\Sigma }_i$. To localize neighboring robots, dead-reckoning predictions are combined with visual measurements using Kalman filtering:

15 $$\begin{aligned} \textbf{K}= & \boldsymbol{\Sigma }_{ij}^{DR} (\boldsymbol{\Sigma }_{ij}^{DR} + \textbf{R}_{visual})^{-1} \end{aligned}$$

16 $$\begin{aligned} \hat{\textbf{r}}_{ij}= & \hat{\textbf{r}}_{ij}^{DR} + \textbf{K}(\textbf{z}_{visual} - \hat{\textbf{r}}_{ij}^{DR}) \end{aligned}$$

where $\textbf{K}$ is Kalman gain, $\hat{\textbf{r}}_{ij}^{DR}$ is dead-reckoning prediction, and $\textbf{z}_{visual}$ is visual measurement^40^.

Cross-modal data association determines correspondence between acoustic and visual observations using Mahalanobis distance:

17 $$\begin{aligned} d^2 = (\textbf{p}_{audio} - \textbf{p}_{visual})^T (\boldsymbol{\Sigma }_{audio} + \boldsymbol{\Sigma }_{visual})^{-1} (\textbf{p}_{audio} - \textbf{p}_{visual}) \end{aligned}$$

Association declared when $d^2 < \chi ^2_{0.95,2} = 5.99$. Successfully associated observations fused using covariance intersection:

18 $$\begin{aligned} \boldsymbol{\Sigma }_{fused}^{-1} = \omega \boldsymbol{\Sigma }_{audio}^{-1} + (1-\omega )\boldsymbol{\Sigma }_{visual}^{-1} \end{aligned}$$

with $\omega \in [0,1]$ minimizing fused covariance trace^41^.

**Fig. 6:**
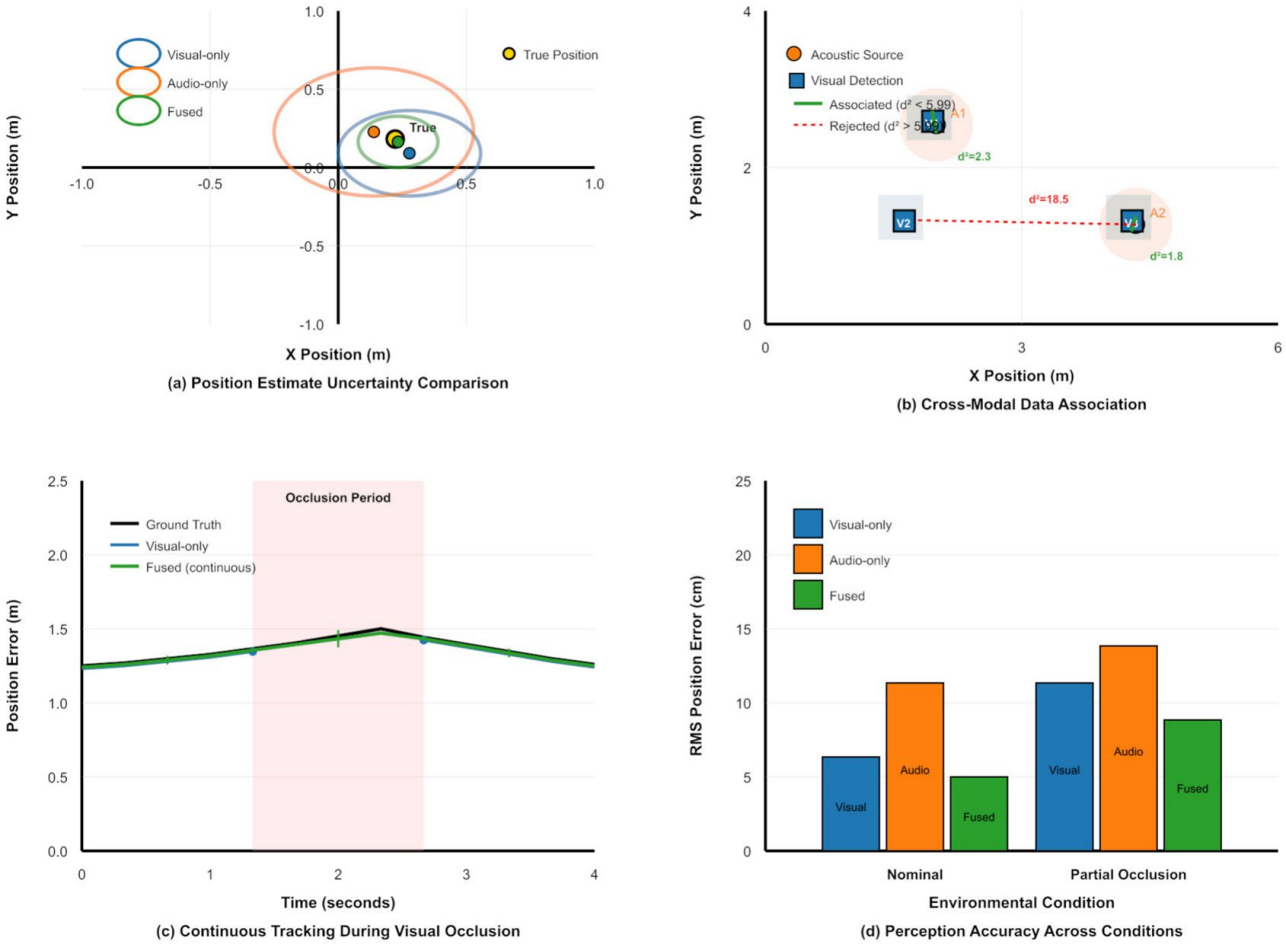
Multi-modal sensor fusion and integrated perception capabilities. (**a**) Position estimate uncertainty ellipses comparing visual-only, audio-only, and fused estimates, demonstrating reduced uncertainty through fusion. (**b**) Cross-modal data association example showing acoustic source location estimate overlaid with visual robot detections, with Mahalanobis distance metrics computed for association decisions. (**c**) Continuous neighbor tracking performance during visual occlusion events, showing fused estimate maintaining accuracy through dead-reckoning and acoustic cues. (**d**) Perception accuracy comparison across sensing modalities for varying environmental conditions (nominal, partial occlusion, high acoustic noise), showing fusion robustness.

Figure 6 illustrates sensor fusion performance benefits. Fused position estimates achieve 35% lower uncertainty compared to visual-only estimates and 60% improvement over audio-only estimates. Cross-modal association successfully identifies 89% of cases where acoustic sources correspond to visually observed robots. The integrated perception system maintains continuous neighbor tracking even during temporary visual occlusions lasting up to 2 seconds through dead-reckoning supported by acoustic observations.

### Experimental validation and field performance

A controlled indoor lab measuring 8 × 12 m was used for the testing, along with an outdoor patio measuring 15 × 20 m. OptiTrack motion capture is an option for capturing ground truth and outdoor manual measurements. The experimental outcomes encompass initialisation of sizes ranging from 3 to 12, maintaining their behavior while in motion, and adapting to cluttered obstacles to ensure reliable communication links and reduce power usage. The failure of the Equipment resulted in the exclusion of 5% of the configuration tested in more than 10 instances, which meant that all necessary periodic assessments were not carried out. More than 300 genuine experiments were included in each experimental campaign^42^.

A metric called root-mean-square error has been found to be useful in characterising formation control. During convergence formation, there were errors of 12.3 cm, 14.7 cm, and 13.9 cm in 8 robots at a speed of 0.3 m/s, and complexity ranges from 8 to 12. It was able to track the trajectory accurately within 25 cm of the reference trajectory during successful 5-min missions. The scalability evaluation revealed a gradual decrease in formation error as unit formations increased, with a 5% decrease for 3 to 12 units.

The assessment of adaptive behavior was based on the delivery of acoustic stimuli (2 kHz pure tones) and visual targets through performance of formation missions. The formation is adjusted to match sound sources and is recalculated in 3–5 s without sacrificing any of its geometries. Deformed formations were shown to be effective in preventing the detection of obstacles with a clearance margin of at least 50 cm. Indoors have a lower latency of 97% compared to the outside, resulting in higher reliability of communication.

The adoption of distributed sensing resulted in 8.2 W per robot for energy efficiency during development missions, whereas the centralized sensing system used with less than 12.5 W consumed 34% less energy. The mission’s duration was increased from a central operation of 67 min to a distributed 91-min operation. Discharging of the battery was consistent across the organization, as indicated by the battery’s voltage. Calculation and communication are both viable options^43^.

**Fig. 7:**
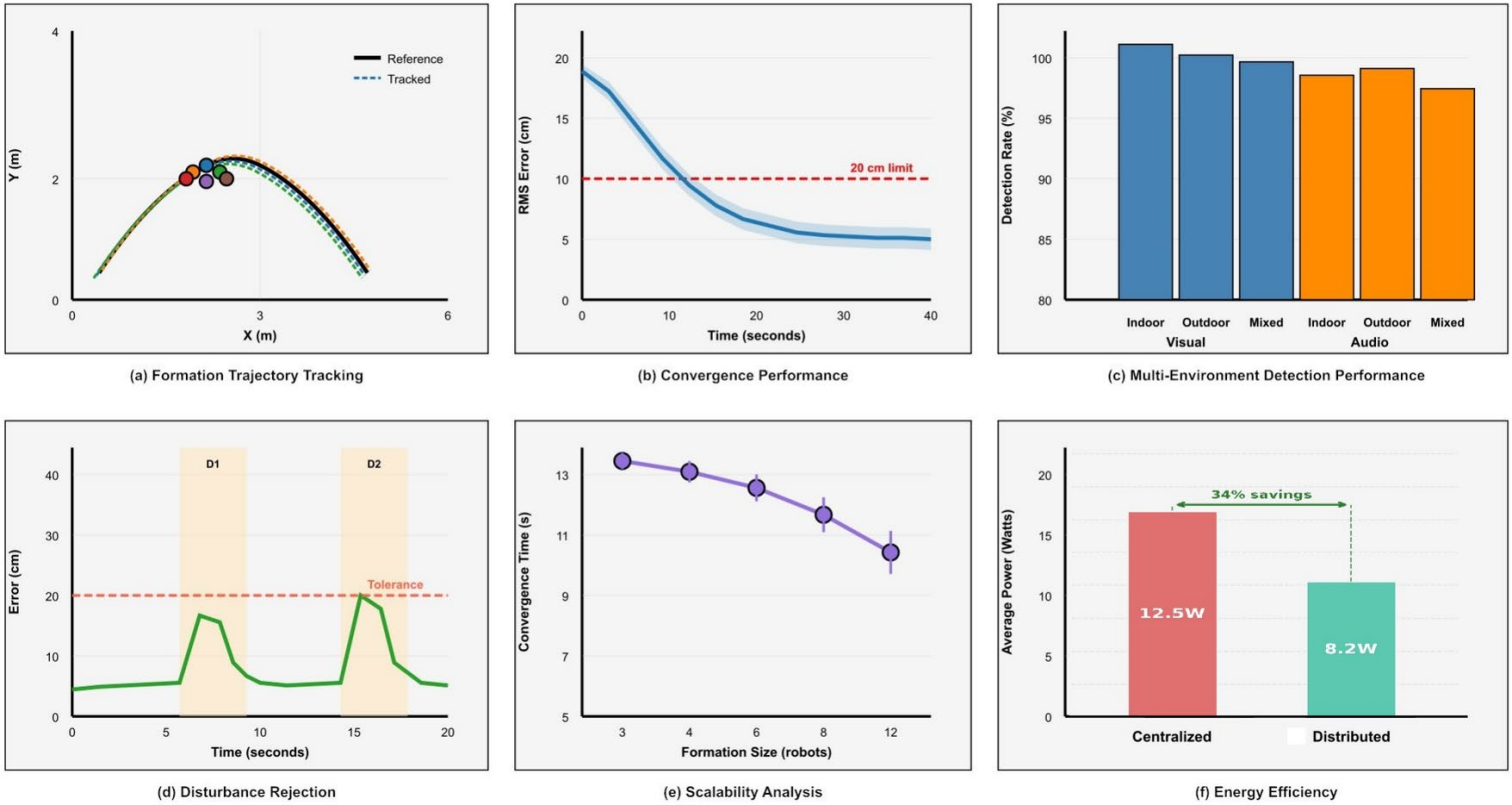
Comprehensive experimental validation and field performance results. (**a**) Formation trajectories for 8-robot wedge formation executing figure-eight path, with motion capture ground truth (solid) and estimated trajectories (dashed) showing excellent tracking performance. (**b**) Formation error statistics for three formation types and multiple sizes, showing mean, standard deviation, and maximum errors maintaining below 20 cm threshold. (**c**) The adaptive formation reconfiguration sequence (t = 0s, 2s, 4s, and 6s) in reaction to acoustic source detection in which the tumescent drive vector changes its course by turns toward the active source, leaving behind one other drone each time. (**d**) Power consumption profiles comparing distributed and centralized sensing architectures over 60-min mission, showing 34% energy reduction for distributed approach. (**e**) Scalability analysis showing formation control performance metrics versus formation size for 3–12 robots. (**f**) Robustness evaluation under communication dropout conditions, demonstrating graceful degradation and recovery capabilities.

Figure 7 presents detailed experimental validation results that demonstrate the performance of the system across different metrics and operational scenarios. The findings imply that ESP32s with limited resources can be utilized for adaptive multi-robot formation control with the aid of embedded audio-visual sensing and Bluetooth communication.

**Fig. 8:**
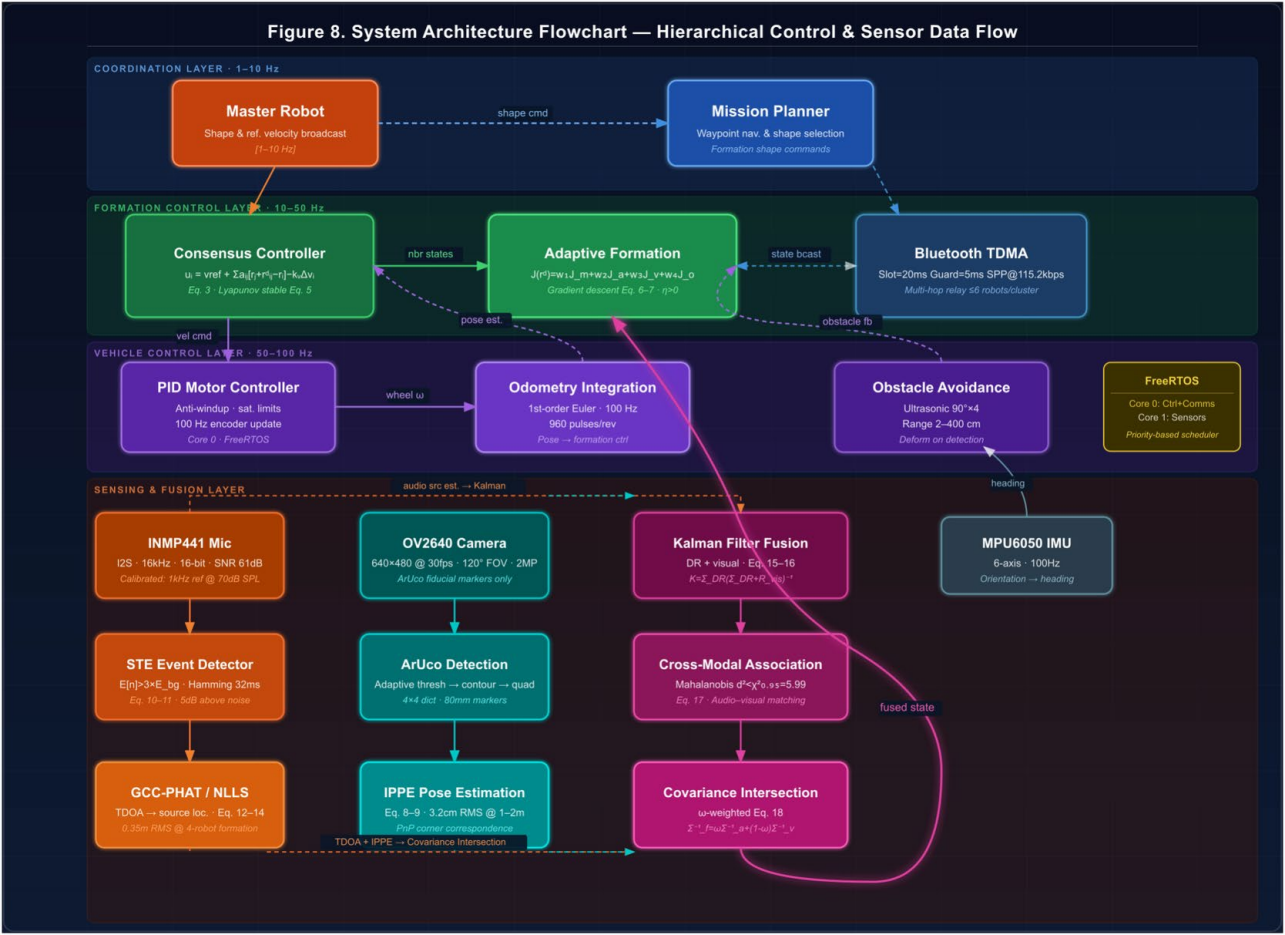
System architecture flowchart: hierarchical control and sensor data flow. The diagram illustrates the four-layer architecture: (top) Coordination Layer—the master robot broadcasts reference velocity and formation shape commands at 1–10 Hz, supported by a mission planner for waypoint navigation; (second) Formation Control Layer—a consensus controller (Eq. 3) and adaptive formation optimizer (Eqs. 6–7) run at 10–50 Hz on each robot, communicating neighbor states via Bluetooth TDMA; (third) Vehicle Control Layer—PID motor controllers, odometry integration, and ultrasonic obstacle avoidance execute at 50–100 Hz under FreeRTOS dual-core scheduling; (bottom) Sensing & Fusion Layer—INMP441 microphone, OV2640 camera, and MPU6050 IMU provide raw observations that are processed through STE event detection, GCC-PHAT/NLLS acoustic localization, ArUco detection, IPPE pose estimation, and finally fused via Kalman filtering, cross-modal association (Eq. 17), and covariance intersection (Eq. 18) before being fed back to the formation control layer.

## Discussion and conclusion

The experiment outcomes revealed the impressive combination of distributed audio-visual sensing and Bluetooth communication networks in a multi-robot formation control scheme on ESP32 platforms using low-resource platforms. The 15 cm RMS error in maintenance of the formation is good enough for applications not demanding centimeter-level performance like environmental monitoring, cooperative transportation, and distributed surveillance. In some situations, other sensing mechanisms such as ultra-wideband ranging or external positioning infrastructure implementation can result in better accuracy.

The use of hierarchical control systems provides advantages such as the ability to be modular, less complicated for stability analysis, and a clear-cut approach to identifying problems. The dual-core ESP32 architecture of each robot supports local formation control, sensor processing, and state estimation, which can be distributed in a semi-distributable manner. Additionally, a centralized coordination layer on the master robot sends out formation shape commands and reference velocities at 1–10 Hz to minimize communication overhead. The integration of consensus based formation control with connectedness of communication graphs has resulted in a decrease in the need for adjustment of edge weights and an increase in damping orders. The formation geometry and adaptive design system is a function of environmental factors, but there is no guarantee that the global formations generated under gradient-based methods will yield the best results. Future work may involve the use of alternative optimization methods, such as evolutionary and reinforcement learning methods.

The Bluetooth communication platform offers a dependable formation-control service with a propagation delay of less than 100 ms and a rate exceeding 92%. Higher formations are too difficult to scale with IPv6 7 devices on current ESP32. The synchronization of *n=12* robots was achieved through a two-level multi-hop relay topology that kept within the per-device connection limit of the swarm (see “Communication Protocol and Network Performance” section). It is likely that using WiFi mesh networks or mixed Bluetooth-WiFi networks would be more effective in facilitating the deployment of large swarms of robots, which are likely to exceed 12 in number. The TDMA protocol incorporates flexible latency gaps that are essential for control stability, but it also curtails throughput, which safeguards the audio signal rate and visual aesthetics.

The proposed sensor fusion approach employs a weighted combination of audio and video modes, resulting in better results than single-use sensors with potential limitations. Before fusion, audio and video are processed separately; a possible alternative is to use flash recommendations for audio channels to refocus visual elements. Enables the use of ArUco fiducials, which enables the detection of visual markers up to 3 m away, but necessitates a sight line between robots. The obstruction of line-of-sight by columns is a significant problem for vision-based localization. The system currently manages occlusions by using dead-reckoning between visual updates, but when these events occur at a distance, drift errors occur. Ultra wideband ranging and other methods of relative localization could also be utilized to resolve this issue. Moreover, the computational complexity of visual processing on ESP32 platforms restricts frame rates to 15–20 fps, which could potentially hinder high-speed formation maneuvers.

The acoustic source localization accuracy for formation sizes of 4 to 8 robots (35 cm RMS) is reasonable enough for sound source detection and approximate localization, but only to a certain extent for precise tracking. The accuracy of localization at ultrasonic frequencies is limited by a finite wavelength and time synchronization accuracy of ±5 ms. This new version is built on a GCC-PHAT based TDOA system that is simplistic and works well in anechoic/slightly reverberating environment, but performs poorly in more challenging indoor environments. Room acoustics simulations or machine learning algorithms may be utilized to enhance the effects of reverberation in more advanced techniques.

Changes in environmental lighting can distort visual observations, while contamination from wind noise and faulty acoustic receptions can lead to formation presses. AABB’s differential-driven system prevents wheel slip, but it experiences difficulties on slippery or icy terrain and requires advanced traction control. The lack of joint charge units necessitates training robots to ensure they have enough battery life during their missions. The device has not been tested in various practical scenarios, including multiple robot joints, dynamic obstacle avoidance maneuvers, and adversary conditions such as sensor attacks (spoofing) and noise based jamming. Testing for long-duration autonomy can be used to establish the reliability of components over extended periods, just as running for reliability during long-term use can help identify failure modes and maintenance intervals.

Field experiments in application areas would outpace laboratory experiments, and this work is a precursor to integration with more complex multi-agent architectures. The ESP32 platform, which has been validated as a physical execution layer for its low cost, could facilitate compelling abstractions like Distributed Multi-Agent Digital Twins^44^ and safety-enforced distributed formation control^45^. Future work will focus on narrowing the gap between the hardware platform and enabling real-time digital twin synchronization and formally validated formation controllers.

## Data Availability

The datasets generated and analyzed during the current study, including raw experimental data, robot firmware source code, and analysis scripts, are available from the author upon reasonable request.
